# SLC6A14 Depletion Contributes to Amino Acid Starvation to Suppress EMT-Induced Metastasis in Gastric Cancer by Perturbing the PI3K/AKT/mTORC1 Pathway

**DOI:** 10.1155/2022/7850658

**Published:** 2022-07-12

**Authors:** Qie Guo, Wen Xu, Xiao Li, Jia-Lin Sun, Xiao-Ce Gu, Fan-Bo Jing

**Affiliations:** ^1^Department of Clinical Pharmacy, The Affiliated Hospital of Qingdao University, Qingdao, Shandong 266003, China; ^2^Department of Clinical Pharmacy, People's Liberation Army 401 Hospital, Qingdao, China

## Abstract

Metastasis is the main obstacle for the treatment of gastric cancer (GC), leading to low survival rate and adverse outcomes in CG patients. SLC6A14, a general amino acid transporter, can import all the essential amino acids in a manner dependent on the NaCl-generated osmotic gradients. Herein, we constructed GC cell sublines with high (SGC7901-M and MKN28-M) and low (MKN28-NM and SGC7901-NM) metastatic ability. Putative functional genes advancing GC metastasis were identified using mRNA microarray analysis and High-Content Screening. In particular, most significant change with a dampening trend in the migration potentiality of GC cells emerged after SLC6A14 gene was silenced. SLC6A14 expression was positively correlated with the migrated capability of different GC cell lines, and SLC6A14 was also constitutively expressed in GC patients with venous or lymphatic invasion, lymph node, or distant metastasis and poor prognosis, thus prompting SLC6A14 as a nonnegligible presence in supporting GC migration and invasion. Consistently, SLC6A14 depletion drastically depressed GC metastasis *in vitro* and *in vivo*. Most importantly, pharmacological blockade and gene silence of SLC6A14 both restricted epithelial-mesenchymal transition- (EMT-) driven GC metastasis, in which attenuated activation of the PI3K/AKT/mTORC1 pathway caused by amino acid starvation was involved. In summary, it is conceivable that targeting SLC6A14 has a tremendous promising for the treatment of metastatic GC.

## 1. Introduction

Gastric cancer (GC) remains one of the most commonly diagnosed cancers of human beings with high incidence and high-grade malignancy [1]. Since its insidious onset, GC usually gets dropped, and most GC patients are diagnosed at an advanced stage for that tumor metastasis is common [2]. Tumor metastasis represented a substantial contributor for the unfavorable prognosis in GC patients and the majority of GC deaths [3]. A median survival period has reportedly ranged only from 3 to 6 months in patients with metastatic GC [4]. Metastasis management for GC is imperative, and this assigns clinical doctors a persistent challenge.

Epithelial-mesenchymal transition (EMT) refers to a well-established process that epithelial cells lose intercellular “self-discipline” and are provided with mesenchymal features [5]. E-cadherin decrease and the upregulation of N-cadherin, Vimentin, and *α*-SMA characterize the EMT initiation [6]. Transcription factors such as Twist1, Slug, ZEB1, and ZEB2 have been proved to regulate the expression of these EMT markers [7]. Abundant evidences have revealed that EMT is a key mechanism participating in GC metastasis [8–10]. Target mining for novel biomarkers prompting GC EMT and metastasis is urgently required, leaving us lots of to elucidate.

As acknowledged, a steady influx of exogenous nutrients is essential for standing up the aggressive biobehaviors of tumor cells [11]. The well-declared increase in glucose uptake may comprise only one chapter in the story of metabolic reprogramming as tumor cells have a continuous requirement for amino acids as well [12]. Upregulated expression of solute carriers (SLCs), which are newly recognized amino acid transporters, holds a great potential to open up a unique peculiarity in tumor cells termed “amino acid addiction” [13]. SLC6A14, also known as ATB^0,+^, is special among the SLC family and exhibits distinctive properties which distinguish it from other SLCs. SLC6A14 transports 18 of the 20 proteinogenic amino acids except glutamate and aspartate in an Na^+^- and Cl^−^-dependent manner, making the transportation almost unidirectional [14]. It is fairly confident that SLC6A14 has a purchasing power of concentrating its substrates more than 1000 times inside cells [15]. Recently, the role of SLC6A14 in fueling cell proliferation and carcinogenesis has been well documented in colorectal cancer [16], thyroid cancer [17], estrogen receptor-positive breast cancer [18], and pancreatic cancer [19]. However, much is unknown about the implications of SLC6A14 in GC malignancy, whose predictions for GC treatment are also not fully explicated.

Here, we established GC cell sublines with high (MKN28-M and SGC7901-M) and low (MKN28-NM and SGC7901-NM) metastatic ability. The differentially expressed genes (DEGs) in MKN28-M and SGC7901-M, compared with MKN28-NM and SGC790-NM cells, respectively, were screened using mRNA microarray analysis. Among these DEGs, 10 upregulated transcripts with a fold change ≥ 3 in the mRNA expression in both MKN28-M and SGC7901-M cells have attracted extensive attentions. Of these 10 genes, SLC6A14 was substantiated as a motivation factor in encouraging the migration of GC cells. High expression of SLC6A14 was correlated with improved facility of GC invasion and metastasis and a poor prognosis in GC patients. Pharmacological blockade and gene silence of SLC6A14 enormously wrecked the migrated and invasive ability of GC cells and lessened GC metastasis in tumor-bearing mice. Moreover, SLC6A14 depletion instigated amino acid starvation to weaken the activation of the PI3K/AKT/mTORC1 pathway, thus impeding the EMT development which was considered to be a culprit for GC metastasis. In conclusion, SLC6A14 could be a treated target against metastatic GC.

## 2. Materials and Methods

### 2.1. Cell Lines

Human GC cell lines MGC-803, BGC-823, SGC-7901, and MKN-28; human gastric epithelia cell line GES1; human embryonic kidney 293 (HEK-293); and 293T cells were purchased from the American Type Culture Collection (ATCC). These cells were maintained in Dulbecco's modified Eagle's medium (Sigma-Aldrich) that was supplemented with fetal calf serum (Life Technologies), penicillin (100 U/mL) and streptomycin (100 *μ*g/mL). All the cells were cultured at 37°C in a humidified atmosphere with 5% CO2.

### 2.2. Reagents

Recombinant human insulin-like growth factor-1 (IGF-1) and *α*-methyltryptophan (*α*-MT) were obtained from Cell Signaling Technology (New England BioLabs Inc.).

### 2.3. Quantitative Real-Time PCR Analysis

Total RNA from indicated cells was extracted by the TRizol regent (Invitrogen), and cDNAs were synthesized using SuperScript™ III (Thermo Scientific). The expressions of SLC6A14, asparagine synthetase (ASNS), and C/EBP-homologous protein (CHOP) were determined using real-time PCR analysis with the support of TaqMan™ Master Mixes (Thermo Scientific), according to the manufacturer's procedure. GAPDH and HPRT1, the housekeeping genes, were used as the controls for normalization. PCR primers for SLC6A14, GAPDH, HPRT1, ASNS, and CHOP gene were provided as follows: SLC6A14 (forward: 5′-GAAGGAGAAAGTGTCGGCTTCA-3′; reverse: 5′-TACCACCTTGCCAGACGATTTG-3′), GAPDH (forward: 5′-GAAGGTGAAGGTCGGAGT-3′; reverse: 5′-CATGGGTGGAATCATATTGGAA-3′), HPRT1 (forward: 5′-GCGTCGTTAGCGATGATGAAC-3′; reverse: 5′-CCTCCCATCTCCTTCATGACATCT-3′), ASNS (forward: 5′-GCACGCCCTCTATGACAATG-3′; reverse: 5′-CTCACTCTCCTCGGCTTT-3′), and CHOP (forward: 5′-GAGAACCAGGAAACGGAAAC-3′; reverse: 5′-GCAGATTCACCATTCGGTC-3′).

### 2.4. Isolation of High-Metastatic and Low-Metastatic GC Sublines

SGC-7901 and MKN-28 cells were suspended in a six-well simplified Boyden chamber with an 8-micron polyethylene terephthalate (PET) membrane (Trevigen, USA). Adherent cells were serum-starved, and the bottom chamber was consumed with Tissue Culture Growth Media (1.5 mL) which contains 20% heat-inactivated fetal bovine serum (FBS). After incubation for 24 h at 37°C, the migrated cells on the bottom chamber and the nonmigrated cells on the top chamber were aspirated carefully. These harvested cells were cultured and passaged aseptically. Above steps were repeated until 15-round selection was completed. These cells that failed to migrate through the chamber were characterized as low-metastatic GC sublines (MKN28-NM and SGC7901-NM), and the GC sublines that located into the bottom chamber were named as MKN28-M and SGC7901-M with high metastatic ability.

### 2.5. Western Blot Analysis

Total proteins from indicated cells or tissue specimens were prepared using RIPA Lysis Buffer (Solarbio Science & Technology Co., Ltd., Beijing, China). Equal amount of protein samples was separated by 12% SDS-PAGE gels and transferred to the nitrocellulose membranes (Millipore, Bedford, USA). The membranes were blocked in Tris-buffered saline with 5% (*w*/*v*) nonfat dry milk and then cultured with primary antibodies overnight at 4°C, followed by the incubation with Goat Anti-Rabbit IgG H&L (HRP) (1 : 100 dilution, ab6721, Abcam) for 1 h at room temperature. Immunoreactive proteins were visualized using ChemiDoc™ XRS^+^System (Bio-Rad) with the help of IMMOBILON WESTERN CHEMILUM HRP SUBSTRATE (Millipore). Relative intensities of indicated proteins towards *β*-actin were estimated. Detailed information for primary antibodies were shown as follows: rabbit anti-human SLC6A14(ab254786), anti-human ZEB2(ab223688), anti-human Twist1 (ab175430), and anti-human PI3K (p85*α*, ab191606) mAbs from Abcam; rabbit anti-human *β*-actin (#4970), anti-human AKT (#4685), anti-human mTOR (#2983), anti-human GSK-3*β* (#12456), anti-human phospho-AKT (#9018), anti-human phospho-GSK-3*β* (#5558), anti-human phospho-mTOR (#5536), anti-human N-cadherin (#13116), anti-human Vimentin (#5741), anti-human E-cadherin (#14472), anti-human mLST8 (#3274), anti-human PRAS40 (#2610), anti-human 4EBP1 (#9644), anti-human Phospho-4E-BP1 (Thr37/46) (#2855), anti-human p70S6K (#2708), anti-human Phospho-p70S6K (Thr421/Ser424) (#9204), anti-humanPhospho-Raptor (Ser792) (#2083), anti-human Phospho-PRAS40 (Thr246) (#2997), and anti-human *α*-SMA (#19245) mAbs from Cell Signaling Technology; and rabbit anti-human Phospho-p85 (Tyr458)/p55 (Tyr199) (PL0304731) from PLLABS (British Columbia).

### 2.6. mRNA Microarray Analysis

Total RNA from high-metastatic (MKN28-M and SGC7901-M) and low-metastatic (MKN28-NM and SGC7901-NM) GC cells was extracted using Cells-to-CT Kit (Thermo Fisher Scientific), according to the manufacturer's instructions. All the RNA was quantified by a UV spectrophotometer (Beckman Coulter, Brea, CA, USA), and RNA integrity numbers were inspected by Agilent Bioanalyzer 2000 (Agilent). mRNA expression profiles for individuals were confirmed using human Clariom™ S Assay platform (Affymetrix). RNA samples were employed with the primers containing a T7 promoter and executed by reverse transcription reaction for the generation of single-stranded cDNA (ss-cDNA). 3′Adaptor was added to ss-cDNA, which was further converted to complementary RNA (cRNA) by *in vitro* transcription (IVT) amplification. CRNA was then converted to biotinylated double-stranded cDNA (ds-cDNA) using GeneAtlas® Hybridization Station (Affymetrix). Arrays were washed and stained using Affymetrix GeneChip Fluidics Station 450 systems, followed by the scanning with GeneChip® Scanner 3000 7G (Affymetrix). Differentially expressed genes (DEGs) (fold change ≥ 1.5 and^∗^*P* < 0.05) were generated using R package.

### 2.7. Pathway Annotation and Enrichment Analysis

Total RNA was isolated from MKN28-M cells that were transfected with LV-pTZU-SLC6A14 or LV-pTZU6+1 plasmid for 48 h. DEGs in MKN28-M cells with SLC6A14 silence were screened using mRNA microarray analysis via the Prime View Human GeneChip and GeneChip Hybridization Wash and Stain Kit (Agilent). These DEGs were analyzed through NCBI Gene Expression Omnibus Software based on the GSEA database. KEGG (Kyoto Encyclopedia of Genes and Genomes) pathway analysis for these DEGs was implemented using Bioconductor and STRING 9.1 software. The significance levels of gene enrichment in each pathway were analyzed through Fisher's exact test.

### 2.8. High-Content Screening

MKN28-M cells were seeded into the 48-well plates and transfected with Lentivirus vectors that silenced candidate genes and then prepared for wound healing assay. The 2-day cell migration rate was calculated using the Celigo® Image Cytometer and Cellomics Array Scan System according to the formula described in the *Supplementary Materials and Methods*. The migrated ability of MKN28-M cells was assessed based on the fold change of migration rate (migration rate on day 2 for the group with the treatment of control vector/migration rate on day 2 for the group with the treatment of shRNA vector).

### 2.9. Plasmid Construction

siRNA duplexes targeting the open reading frame of SLC6A14 gene with loop ring (sense-loop-antisense) were designed using BLOCK-iTTM RNAi Designer and then synthesized into oligonucleotides (ODNs) with BamHI and EcoRI overhanging ends by GenePhama Biotech Co., Ltd. (Shanghai, China). Annealed top and bottom chains of single-stranded ODN were mixed, followed by the Polymerase Chain Reaction (PCR) for the generation of shRNA templates. The shRNA templates were cloned into the pTZU6+1 vector using T4 DNA ligase, and the conjugated product was transformed into E. coli DH5*α* with Amp^+^LB cover. The clones with correct insertion of target sequence were named as pTZU-SLC6A14 plasmid.

Alternatively, the coding sequence of SLC6A14 and Raptor gene was searched in Genbank, and PCR primers targeting SLC6A14 and Raptor gene with EcoRI and XhoI overhangs were designed using Primer 5.0 and DNA Star software. SLC6A14 and Raptor cDNA were acquired from HEK-293 cells using PCR amplification. PCR products were cloned into the pcDNA3.1 plasmid for the construction of pcDNA3.1-SLC6A14 and pcDNA3.1-Raptor plasmid.

The recombinant vectors and the packaging plasmids pMD2.G and psPAX2 were transfected into 293 T cells. 48 h later, culture supernatant was collected and filtered through a 0.50 mm filter, followed by the centrifugation (10,000 g) at 4°C for 1 h to capture the viruses containing LV-pTZU-SLC6A14, LV-pcDNA3.1-SLC6A14, and LV-pcDNA3.1-Raptor plasmid.

LV-pTZU-SLC6A14, LV-pcDNA3.1-SLC6A14, and LV-pcDNA3.1-Raptor plasmid were transfected into GC cells using Lentivirus Enhancement Reagent Envirus™-LV (Engreen Biosystem Co, Ltd.) according to the manufacturer's instructions.

### 2.10. Human Tissue Samples

103 GC patients without receiving chemotherapy and radiotherapy but underwent surgical resection were followed up for the evaluation of overall survival (OS) and disease-free survival (DFS) time. Tissue specimens were obtained from these patients, and normal gastric mucosas were taken as the controls. Additionally, 20 paired GC tissues and adjacent normal tissues (ANTs) were kindly provided by the department of pathology in the Affiliated Hospital of Qingdao University. These tissue samples were embedded in paraffin for immunohistochemistry or were stored in liquid nitrogen for Western blot analysis. The Ethics Committee of the Qingdao University approved this study, and full consent for each patient was acquired.

### 2.11. Animals and In Vivo Metastasis Assay

MKN28-M cells were transfected with LV-pSin-hyg-GFP/luc2 plasmid (GenePhama, Shanghai, China) via JetPEI reagent (PolyPlus-transfection FARANCE), followed by the Hygromycin (Sigma-Aldrich) (300 *μ*g/mL) administration for the collection of luciferase-labeled MKN28-M cells. These luciferase-labeled MKN28-M cells were then transfected with LV-pTZU-SLC6A14 (MOI = 60) or LV-pcDNA3.1-SLC6A14 (MOI = 60) plasmid and were exposed for two weeks to Puromycin (2.5 *μ*g/mL). BALB/C nude mice (6-8 weeks old) were purchased from Vital River Laboratory Animal Technology Co., Ltd (Beijing, China) and were kept under specific pathogen-free (SPF) conditions. The tumor-bearing mice were randomly divided into four groups as follows: (1) control group, which was injected with luciferase-labeled MKN28-M cells via tail vein; (2) *α*-MT treatment group, for which *α*-MT (3 mg/mL/kg) was prepared in drinking sucrose water and then continuously administered 3 days prior to intravenous injection of luciferase-labeled MKN28-M cells; (3) SLC6A14 silence group, which was injected intravenously with luciferase-labeled MKN28-M cells after the transfection of LV-pTZU-SLC6A14 plasmid; and (4) SLC6A14 overexpression group, which was injected via tail vein with luciferase-labeled MKN28-M cells after the transfection of LV-pcDNA3.1-SLC6A14 plasmid. The mice in each group were injected intraperitoneally with D-luciferin (Invitrogen) (120 mg/kg) and were observed once a week since cell injection using the Imaging Platform for Small Animals in Vivo IVIS® Lumina III (PerkinElmer). All experimental studies were approved by the Qingdao University Animal Care and Use Committee.

### 2.12. Statistical Analyses

Statistical analysis was performed using a paired Student *t*-test, *χ*^2^ test, ANOVA, and Mann–Whitney *U* test. The association between SLC6A14 expression and the OS or DFS time was evaluated using log-rank test and was demonstrated as the Kaplan-Meier curves. ^∗^*P* < 0.05,  ^∗∗^*P* < 0.01, and^∗∗∗^*P* < 0.001 were considered to be significant.

### 2.13. Wound Healing Assay

Indicated cells were cultured in the 6-well plates until they were assayed to 100% confluence. The bottom of the well was scratched to introduce a gap by a sterile 200 *μ*L pipet tip. Photographs were taken at 0 and 48 h since scratching, and the migration rate was calculated according to the formula shown in the *Supplemental Materials and Methods*.

### 2.14. Transwell Assay In Vitro

Transwell assay was carried out using the Transwell cell culture chamber units (Corning Costar, Cambridge, MA, USA) with an 8-micron polyethylene terephthalate (PET) membrane. Briefly, indicated cells were seeded in the 24-well plates and then starved for 24 h. These cells were reseeded in the top chamber with serum-free medium (180 *μ*L), while culture medium containing 20% FBS was added into the bottom chamber. For the performance of invasion assay, the upper chamber was covered with a film of Matrigel (Trevigen, USA, 200 mg/mL). After incubating for another 48 h, the chamber was take out carefully and washed thoroughly and was fixed in 4% polyformaldehyde for 1 h. Finally, the chamber was stained with 0.1% crystal violet for another 20 min at room temperature and was observed under a microscope.

### 2.15. Immunohistochemistry Assays

Paraffin-embedded tissues were serially sectioned at 4 *μ*m and fixed in Triton X-100 for 10 min; they were then blocked in the mixture of 0.3% BSA and Triton X-100 for 1 h. The sections were then incubated with the primary antibody against SLC6A14 (1 : 500 dilution) at 4°C overnight. After being washed with TBST, tissue slices were incubated with HRP-conjugated secondary antibody (1 : 100 dilution) at room temperature for another 1 h. Finally, these sections were cultured in the diaminobenzidine solution at 37°C for 15 min and were observed under a microscope. Staining scores based on the percentage of positive cells were determined using Image-Pro Plus V6.0 software: Scores 0-1 representing < 10% positive staining (negative, -); Scores 1-2 representing 10-30% positive staining (weak positive, +); Scores 2-3, representing 30-50% positive staining (moderate positive, ++); and Scores 3-5 representing >50% positive staining (strong positive, +++).

## 3. Results

### 3.1. SLC6A14 Is a Candidate Target with a Role in Encouraging GC Metastasis

**Figure 1:**
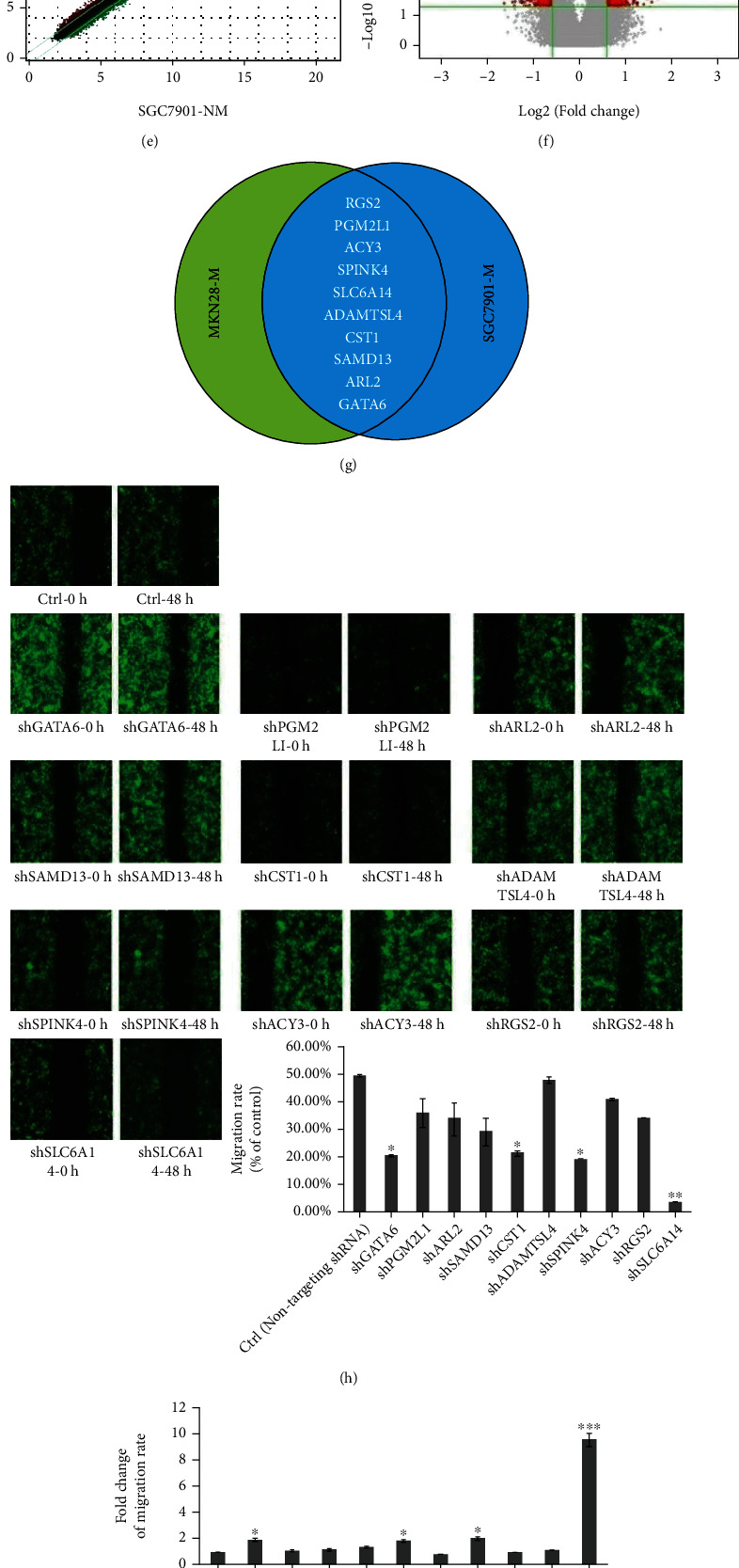
SLC6A14 expression is positively induced in GC cells with strong metastatic ability. mRNA expression profiles in MKN28-M compared with MKN-28-NM (a–c) and in SGC7901-M compared with SGC7901-NM cells (d–f) were confirmed using mRNA microarray analysis. (a, d) Heat map depicting differentially expressed genes (DEGs), in which the upregulated and downregulated genes were represented by red and green columns, respectively (fold change ≥ 1.5 and^∗^*P* < 0.05). (b, e) The scatter plot indicating the distribution of signal intensity between different treatment groups on the rectangular coordinate plane. (c, f) Volcanic maps displaying significant difference were drawn based on the fold change and *P* values. (g) The Venn diagram showing 10 intersectional upregulated genes with a fold change ≥ 3 in their mRNA expressions in both MKN28-M and SGC7901-M, compared with MKN28-NM and SGC7901-NM cells, respectively. (h) Representative images of High-Content shRNA Screening for GATA6, PGM2L1, ARL2, SAMD13, CST1, ADAMTSL4, SPINK4, ACY3, RGS2, and SLC6A14 were obtained from wound healing assay (upper panels). The 2-day migration rate of MKN28-M cells that were transfected with indicated shRNAs was also demonstrated as the means ± SD of three replicates (bottom panel). ^∗^*P* < 0.05 and^∗∗^*P* < 0.01 versus control. (i) Fold change of migration rate in MKN28-M cells with indicated transfections was calculated and was shown as the means ± SD from three independent experiments. ^∗^*P* < 0.05 and^∗∗∗^*P* < 0.001 versus control. (j) SLC6A14 expression in different GC cell lines was checked using Western blot analysis. Data were represented as the strip maps (left panels) and relative gray values towards *β*-actin with the means ± SD from three irrelevant experiments (right panel). ^∗∗^*P* < 0.01 versus the SGC7901-NM and MKN28-NM group. (k) The migration capability of indicated GC cells was evaluated by wound healing assay. Data were demonstrated as the morphology of “wound strack” (left panels) and were also judged as the migration rate with the means ± SD from three triplicate experiments (right panel). ^∗∗^*P* < 0.01 versus the SGC7901-NM and MKN28-NM group.

To find the credible target that got immersed in developing GC metastasis, the differentially expressed genes (DEGs) in high-metastatic (MKN28-M and SGC7901-M) compared with low-metastatic (MKN28-NM and SGC7901-NM) GC cells, were screened using mRNA microarray analysis. Approximately 445 and 487 DEGs exhibiting more than 1.5-fold change were identified in MKN28-M and SGC7901-M compared to MKN28-NM and SGC7901-NM cells, respectively (Figures 1(a)–1(f)). Among these DEGs, 10 upregulated transcripts with a fold change ≥ 3 in the mRNA expression in both MKN28-M and SGC7901-M cells drew lots of attentions, and SLC6A14 was defined as exhibiting the most obvious change in mRNA expression (Figure 1(g), Supplementary Fig. S1A and B). All candidate genes were then silenced in MKN28-M cells, the expression of which concerned with GC metastasis was clarified using High-Content Screening (HCS). Interestingly, SLC6A14 knockdown was gifted with the greatest effect on suppressing the migratory ability of MKN28-M cells (Figures 1(h) and 1(i)). Moreover, MGC-803, BGC-823, SGC7901-M, and MKN28-M had a greater abundance of SLC6A14 protein and also possessed a stronger migratory ability, compared with SGC7901-NM and MKN28-NM cells (Figures 1(j) and 1(k)). It is conceivable that prominent expression of SLC6A14 dominates migrated ability in different GC cell lines. These results strongly promulgate a positive correlation between SLC6A14 expression and GC metastasis.

### 3.2. High Expression of SLC6A14 Is Associated with GC Metastasis and Poor Prognosis in GC Patients

**Figure 2:**
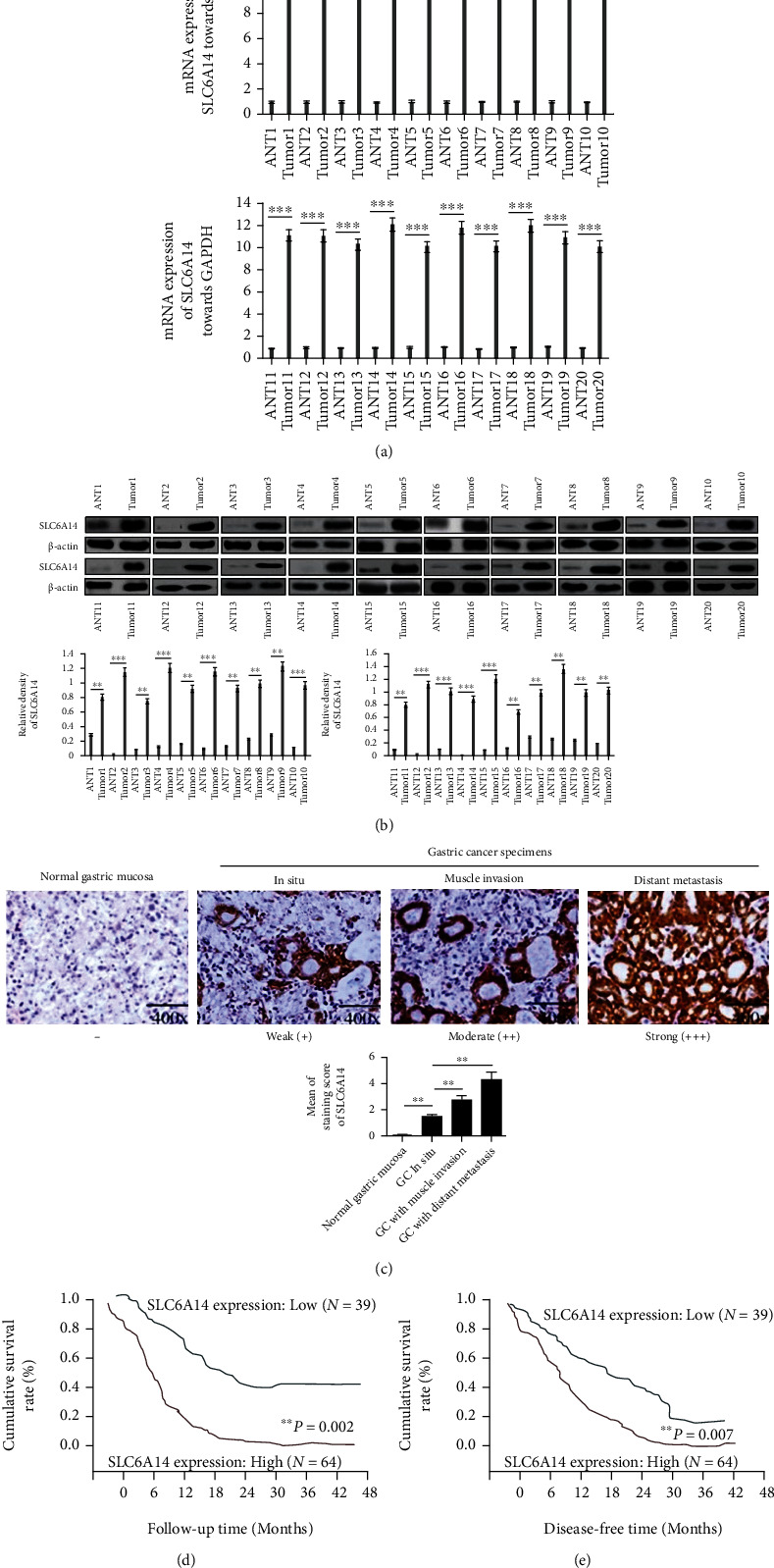
SLC6A14 upregulation is a risk stratification factor for GC patients with metastasis and poor prognosis. (a) Quantitative study of SLC6A14 mRNA in GC and paired ANTs was carried out by Quantitative Real-time PCR analysis. Data are demonstrated as the relative expression towards GAPDH with the means ± SD from three independent experiments. ^∗∗∗^*P* < 0.001. (b) Protein level of SLC6A14 in GC and corresponding ANTs was determined using Western blot assay. Data are shown as the representatives (upper panels) and the relative expression towards *β*-actin with the means ± SD of three replicates (bottom panels). ^∗∗^*P* < 0.01 and^∗∗∗^*P* < 0.001. (c) SLC6A14 expression in serial samples from GC patients was examined by *immunohistochemical staining.* Data are manifested as the representative images (upper panels) and staining scores for SLC6A14 with the means ± SD from three independent experiments (bottom panel). ^∗∗^*P* < 0.01. (d) Overall survival (OS) and (e) disease-free survival (DFS) curves of GC patients were generated using Kaplan-Meier analysis, and the correlation between SLC6A14 expression and OS or DFS time was evaluated by log-rank test. ^∗∗^*P* = 0.002 and^∗∗^*P* = 0.007 were considered to be statistically significant.

To further validate the role of SLC6A14 in touching off GC metastasis, SLC6A14 expression in paired GC tissues and ANTs was assessed. As shown in Figures 2(a) and 2(b), SLC6A14 expression in GC tissues was dramatically exceeded rather than that in the ANTs. Alternatively, tissue samples from GC patients were collected, in which the SLC6A14 expression was evaluated.

Exemplarily data obtained from *IHC assay* demonstrated that GC patients with muscle invasion and distant metastasis exhibited higher SLC6A14 expression as compared with those *in situ* (Figure 2(c)). Notably, SLC6A14 expression was positively correlated with the depth of tumor invasion (^∗^*P* = 0.023), the disposition to distant metastasis (^∗∗^*P* = 0.009), venous invasion (^∗^*P* = 0.033), lymph node metastasis (^∗^*P* = 0.035), and lymphatic invasion (^∗∗^*P* = 0.021) in GC patients (Table 1). Moreover, elevated SLC6A14 expression was also associated with a trend towards shorter overall survival (OS) and disease-free survival (DFS) time in GC patients (Figures 2(d) and 2(e)). These findings give us an enlightenment that SLC6A14 upregulation may be an evil promoter for GC metastasis, probably causing poor prognosis in GC patients.

### 3.3. SLC6A14 Depletion Obstructs GC Metastasis In Vitro and In Vivo

**Figure 3:**
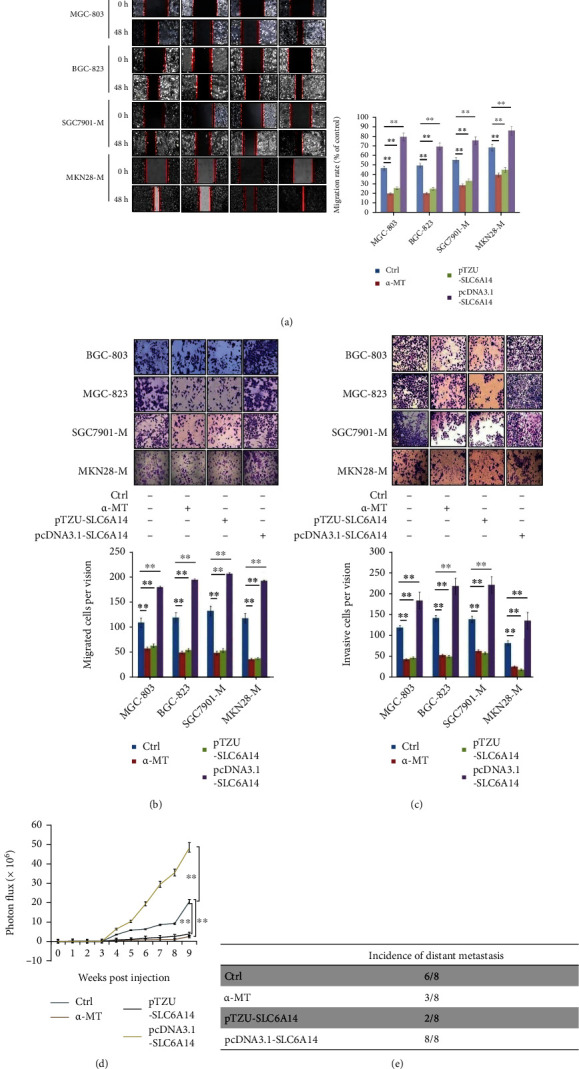
Pharmacological blockage and SLC6A14 silence inhibit GC metastasis. Indicated GC cells were treated with *α*-MT (5 mM), LV-pTZU-SLC6A14 (MOI = 60), or LV-pcDNA3.1-SLC6A14 (MOI = 60) plasmid for 48 h and then prepared for the (a) wound healing, (b) cell migration, and (c) invasion assays. For (a)–(c), data were demonstrated as the representative photographs (left panels in (a) and upper panels in (b, c)) and were also expressed as the migration rate (right panel in (a)) and the number of migrated and invasive cells (bottom panels in (b, c)) with the means ± SD of three replicates. ^∗∗^*P* < 0.01. (d) Luminescent cells in metastasized foci of each group were quantified using IVIS-SPECTRUM *in vivo* photon counting device. Data were shown as the photon flux with the means ± SD from three triplicate experiments. ^∗∗^*P* < 0.01. (e) The number of mice with GC metastasis in each group was summarized.

It followed that aberrant expression of SLC6A14 indicated an increase in the probability of GC metastasis; then, we were particularly interested in ascertaining whether SLC6A14 depletion could constrain GC metastasis. As expected, SLC6A14 depletion with the treatment of *α*-MT and LV-pTZU-SLC6A14 plasmid both remarkably restrained the migratory and invasiveness ability of GC cells, but SLC6A14 overexpression via the transfection of LV-pcDNA3.1-SLC6A14 plasmid distinctly increased the number of migrated and invasive GC cells (Figures 3(a)–3(c)). Moreover, the mice with MKN28-M-based xenografts experienced a limited metastasis to the distance after.

In *α*-MT treatment and SLC6A14 knockdown, however, MKN28-M-injected mice with SLC6A14 overexpression exhibited an increased level of distant metastasis, as shown by the higher *in vivo* bioluminescence signals (Figure 3(d)). Strikingly, distant metastasis incidence in MKN28-M-bearing mice with SLC6A14 depletion was only compared with those of the control group and remaining parts with SLC6A14 overexpression (Figure 3(e)). These data suggest that SLC6A14 plays active roles in guiding GC metastasis, and SLC6A14 depletion can be regarded as a reliable solution in impeding GC metastasis.

### 3.4. SLC6A14 Depletion Induces Amino Acid Starvation to Alleviate the Activation of the PI3K/AKT/mTORC1 Pathway

**Figure 4:**
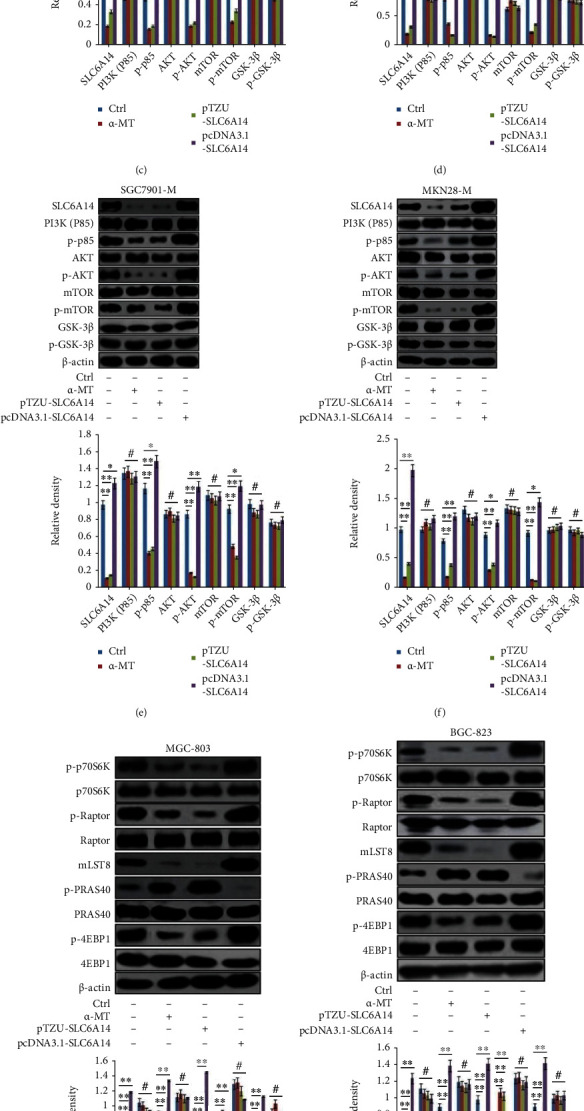
SLC6A14 depletion mediates amino acid starvation to alleviate the activation of the PI3K/AKT/mTORC1 pathway. (a) MKN28-M cells were transfected withLV-pTZU-SLC6A14 plasmid (MOI = 60); 48 h later, differentially expressed genes (DEGs) in SLC6A14-silenced-MKN28-M, compared to MKN28-M cells, were identified using mRNA microarray analysis and were shown in heat map. (b) KEGG pathway analysis was carried out to clarify the top 10 overrepresented canonical pathways that were enriched by these DEGs in MKN28-M cells following LV-pTZU-SLC6A14 transfection. (c–f) Indicated cells were treated with *α*-MT (5 mM), LV-pTZU-SLC6A14 (MOI = 60), or LV-pcDNA3.1-SLC6A14 (MOI = 60) plasmid for 48 h. Western blot analysis was conducted to detect the expression of signaling proteins in the PI3K/AKT pathway in (c) MGC-803, (d) BGC-823, (e) SGC7901-M, and (f) MKN28-M cells, respectively. (g–j) Indicated cells were treated in accordance with the methods as described in (c–f); the expression of mLST8, total, and phospho-Raptor, phospho-PRAS40, phospho-p70S6K, and phospho-4EBP1 was determined by Western blot analysis. For (c–j), the experiments were repeated for three times, and representative results were described as the representatives (upper panels) and the means ± SD of relative expression (bottom panels). ^∗^*P* < 0.05,  ^∗∗^*P* < 0.01, and^#^*P* > 0.05. (k, l) GC cells were stimulated with *α*-MT (5 mM), LV-pTZU-SLC6A14 (MOI = 60), or LV-pcDNA3.1-SLC6A14 (MOI = 60) plasmid for 48 h. (k) ASNS and (l) CHOP mRNA were examined by Quantitative Real-time PCR assay. Data were displayed as the relative expression that were normalized to HPRT1 with the means ± SD from three irrelevant experiments. ^∗∗^*P* < 0.01.

To expound the underlining mechanism by which SLC6A14 promoted GC metastasis, the DEGs in SLC6A14-silenced-MKN28-M, compared to MKN28-M cells, were examined using the Prime View Human GeneChip. 28 upregulated and 70 downregulated clusters were presented in MKN28-M cells with the transfection of LV-pTZU-SLC6A14 plasmid (Figure 4(a)). KEGG pathway analysis verified that these DEGs were primarily enriched in the PI3K signaling pathway (Figure 4(b)). Enrichment analysis detailed that the PI3K signaling pathway in MKN28-M cells with SLC6A14 silence was also enriched by PIK3R1, AKT1, MTOR, etc. (Supplementary Fig. S2). Western blot assay further affirmed that *α*-MT and LV-pTZU-SLC6A14 treatment both markedly suppressed the expression of p-PI3K (P85), p-AKT, and p-mTOR; however, SLC6A14 overexpression considerably increased the protein level of p-PI3K (P85), p-AKT, and p-mTOR in GC cells (Figures 4(c)–4(f)). mTOR binds to different adaptor proteins hence evolving into the mTOR complex (mTORC), including the mTORCl and mTORC2. Among them, mTORCl which consists of the core subunits mTOR, regulatory-associated protein of mTOR (Raptor), mammalian lethal with SEC13 protein 8 (mLST8), and the endogenous inhibitor of the complex, 40 kDa Proline-rich Akt substrate (PRAS40) is thoroughly studied [20, 21]. Through transcriptional mechanisms mediated by its substrates, including ribosomal protein S6 kinase (p70S6K) and eukaryotic translation initiation factor 4E-binding protein1 (4E-BP1), mTORC1 has been discovered to be a central moderator in tumor metabolism activated downstream of PI3K [22]. In line with this, the DEGs covered in the PI3K signaling pathway in MKN28-M cells with SLC6A14 knockdown also included Raptor, PRAS40, mLST8, p70S6K, and 4E-BP1 (Supplementary Fig. S2). Furthermore, p-Raptor, mLST8, p-p70S6K, and p-4EBP1 expressions were decreased profoundly, but p-PRAS40 expression was evidently increased in GC cells with SLC6A14 depletion (Figures 4(g)–4(j)). SLC6A14 overexpression upregulated the protein level of p-Raptor, mLST8, p-p70S6K, and p-4EBP1, but downregulated the p-PRAS40 expression (Figures 4(g)–4(j)). So we can infer that SLC6A14 depletion incites the abated activation of the PI3K/AKT/mTORC1 pathway.

Strong evidences have authenticated that the delivery of the mTORC1 pathway is essential for nutrients intake by tumor cells [23, 24], and amino acid starvation entraps the inactivation of this pathway [25]. To inquire into whether the retardation of the PI3K/AKT/mTORC1 pathway approached by SLC6A14 depletion was attributed to amino acid starvation, the expressions of ASNS and CHOP were then determined because the increased expression of these two was recognized as a hallmark of amino acid deficiency [26]. As shown in Figures 4(k) and 4(l), *α*-MT and LV-pTZU-SLC6A14 administration both augmented the steady-state mRNA level of ASNS and CHOP in GC cells. Collectively, SLC6A14 depletion animates amino acid starvation to extinguish the activation of the PI3K/AKT/mTORC1 pathway.

### 3.5. SLC6A14 Depletion Restrained the EMT Process to Interfere GC Metastasis by Frustrating the Activation of the PI3K/AKT/mTORC1 Pathway

**Figure 5:**
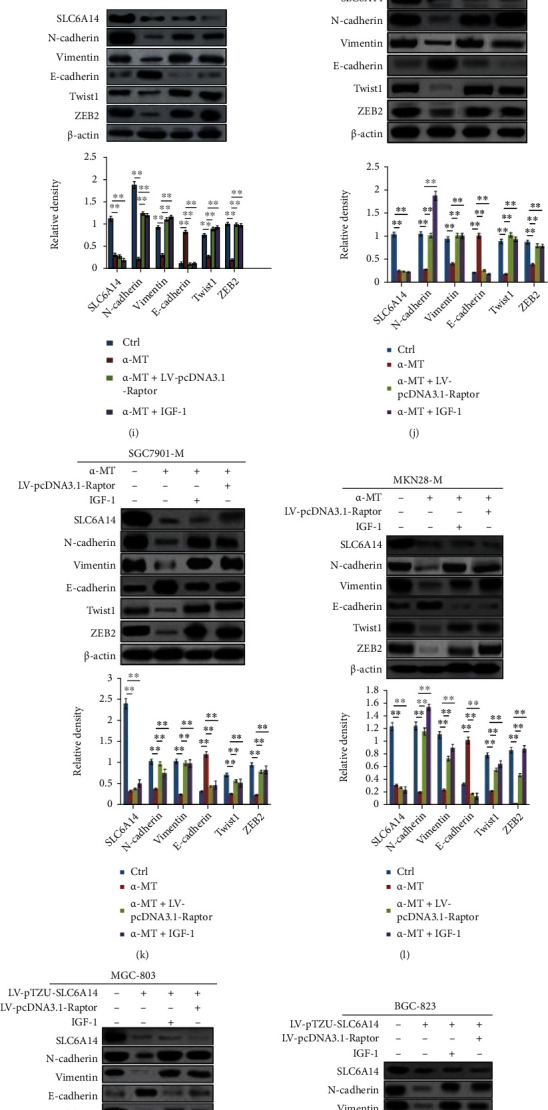
SLC6A14 depletion suppresses the EMT development to delay GC metastasis by attenuating the activation of the PI3K/AKT/mTORC1 pathway. Indicated GC cells were treated with *α*-MT (5 mM) or LV-pTZU-SLC6A14 (MOI = 60) plasmid for 48 h; the protein levels of N-cadherin, Vimentin, E-cadherin, and *α*-SMA in MGC-803 (a), BGC-823 (b), SGC7901-M (c), and MKN28-M cells (d) were checked by Western blot analysis. (e–h) GC cells were stimulated with *α*-MT (5 mM) or LV-pTZU-SLC6A14 plasmid (MOI = 60); 48 hours later, Twist1, ZEB1, ZEB2, and Slug expressions in MGC-803 (e), BGC-823 (f), SGC7901-M (g), and MKN28-M cells (h) were confirmed using Western blot assay. (i–l) Indicated GC cells were prestimulated with IGF-1 (10 ng/mL) for 16 h or were pretransfected with LV-pcDNA3.1-Raptor plasmid (MOI = 60) for 24 h, followed by the *α*-MT treatment (5 mM) for another 48 h. N-cadherin, Vimentin, E-cadherin, Twist1, and ZEB2 expressions in MGC-803 (i), BGC-823 (j), SGC7901-M (k), and MKN28-M cells (l) were detected by Western blot analysis. (m–p) Indicated GC cells were pretreated with IGF-1 (10 ng/mL) for 16 h or were pretransfected with LV-pcDNA3.1-Raptor plasmid (MOI = 60) for 24 h and then transfected with LV-pTZU-SLC6A14 (MOI = 60) plasmid for another 48 h. N-cadherin, Vimentin, E-cadherin, ZEB2, and Twist1 expressions in MGC-803 (m), BGC-823 (n), SGC7901-M (o), and MKN28-M cells (p) were estimated by Western blot analysis. (q, r) Indicated cells were administrated with IGF-1 (10 ng/mL) for 16 h or were transfected with LV-pcDNA3.1-Raptor plasmid (MOI = 60) for 24 h and then treated with *α*-MT(5 mM) (q) and LV-pTZU-SLC6A14 plasmid (MOI = 60) (r) for another 48 h. Transwell assays were performed to identify the migration ability of GC cells. The results were demonstrated as the representative images (upper panels) and the number of migrated cells with the means ± SD from three irrelevant experiments (bottom panels). ^∗∗^*P* < 0.01. For Western blot analysis, data were demonstrated as the corresponding representatives (upper panels) and the means ± SD of relative expression from triplicate experiments (bottom panels). ^∗∗^*P* < 0.01 and^#^*P* > 0.05.

Then, we explored whether SLC6A14 depletion could mediate the alleviation of the PI3K/AKT/mTORC1 pathway to obstruct the EMT process thus interrupting GC metastasis. As depicted in Figures 5(a)–5(d), pharmacologic blockade and gene silence of SLC6A14 both noticeably decreased the N-cadherin and Vimentin expression, but substantially increased the protein level of E-cadherin. In addition to that, ZEB2 and Twist1, but not Slug and ZEB1, were downregulated appreciably in GC cells with SLC6A14 depletion (Figures 5(e)–5(h)). However, IGF-1 and LV-pcDNA3.1-Raptor plasmid, which were considered as the “active catalysts” of the PI3K/AKT signaling pathway, both offset the regulatory effect of SLC6A14 depletion on the expression of these EMT indicators (Figures 5(i)–5(l) and Figures 5(m)–5(p)). Moreover, the impairment of migration ability in GC cells mediated by SLC6A14 depletion was abolished after IGF-1 treatment and LV-pcDNA3.1-Raptor transfection (Figures 5(q) and 5(r)). Overall, SLC6A14 depletion delays EMT advancement to restrict GC metastasis by attenuating the activation of the PI3K/AKT/mTORC1 pathway.

## 4. Discussion

GC represented the fourth most serious cancer and the third greatest cause of cancer-related death worldwide, especially, ranks second in terms of the cancer-related morbidity and fatality in China [27]. Those patients with advanced GC often expressed poor prognosis for that tumor metastasis was given impetus [28].

A large number of amino acids were ingested as the nutritional sources for catering to the malignant behavior, and various kinds of amino acid transporters equipped carrier channels for amino acid uptake in tumor cells [29]. SLCs, especially the SLC1A5/ASCT2, SLC7A5/LAT1, and SLC7A11/xCT, have been well reported and enjoy high media profiles [30]. SLC1A5 transports the lanine, serine, cysteine, threonine, glutamine, and asparagine [31], and the transfer spectrum of SLC7A5 is composed mostly of neutral amino acids [32], while the substrates of SLC7A11-triggered transportation include only the glutamate and cystine [33]. SLC6A14, the newest member belonging to SLCs, can mediate the uniport of almost all the essential amino acids into cells and is ideal to meet the inordinate demands of tumor cells for amino acids [34].

Actually, the proliferation-promoting role of SLC6A14 has been stated in certain human cancers [15–18]. SLC6A14 knockout mice showed delays in the development of mammary tumors when crossed with the polyoma middle T oncoprotein mouse breast cancer model [35]. Here, SLC6A14 also came into our sight and was first mentioned for its powerful driving force in accelerating the migration and invasion of GC cells. To our knowledge, there was also the first report to propose a proof-of-concept that SLC6A14 upregulation operated in favour of GC invasion and metastasis and predicted a poor prognosis in GC patients. It was consonant that pharmacologic blockade and gene silence of SLC6A14 both suspend GC metastasis *in vitro* and *in vivo.* Therefore, our findings are complementary to those of the predecessors, in which the role of SLC6A14 not only in stirring up tumorigenesis but also in facilitating GC metastasis is revealed.

To date, there is no indication about the activated pathways excited by SLC6A14, much less for the precise mechanisms underlined in GC metastasis mediated by SLC6A14. Herein, our findings were firstly enumerated that SLC6A14 depletion distinctly retarded the activation of the PI3K/AKT/mTOR pathway, which was manifested as the decreased expression of the adaptor proteins constituted the mTORC1 complex and the downregulation of signaling proteins manipulated by mTORC1. SLCs have been viewed as the accurate sensors of amino acids for mTORC1 functioning in tumor cells [36, 37]. Emerging evidences have illustrated that SLC38A9 interacts with Rag GTPase in an amino acid-sensitive and nucleotide-binding dependent manner, thereby rousing the activity of mTORC1 [38, 39]. SLC38A9 downregulation moderates the activation of mTORC1 induced by arginine, suggesting that SLC38A9 enhances mTORC1 activity as an arginine sensor [40].

Here, SLC6A14 depletion provokes amino acid starvation followed by the cripple of the PI3K/AKT/mTORC1 pathway, thus raising the possibility that SLC6A14 can be treated as an amino acid sensor to promote GC metastasis through sensitizing the PI3K/AKT/mTORC1 pathway. However, further efforts should be made to certificate what kind of amino acid sensor can be used for SLC6A14 to activate the PI3K/AKT/mTORC1 pathway.

EMT enables many malignant features of GC, in which metastasis has become the most concerned [41]. The activation of the PI3K/AKT pathway plays a crucial role in the EMT take-off in GC [42, 43]. Activated PI3K and AKT can prevent the entry of GSK-3*β* into cell nucleus, thereby relieving the suppressive effect mediated by GSK-3*β* on the expression of ZEB2 and Slug in GC cells [44–46]. Phosphorylated PI3K and AKT also boosted I*κ*B-*α* degradation and NF-*κ*B phosphorylation, therefore increasing the protein level of E-cadherin and Vimentin but downregulating the N-cadherin expression [47–49]. Here, the EMT flourishing generated by the activation of the PI3K/AKT/mTORC1 pathway is also pivotal for GC metastasis, for which SLC6A14 acts as a creator of a bad precedent. We also put forward a convincing evidence that SLC6A14 depletion perturbs the EMT process to suppress GC metastasis by impeding the activation of the PI3K/AKT/mTORC1 pathway.

## 5. Conclusion

Our findings remarked the point of view that SLC6A14 initiated GC metastasis, thus leading an unsatisfactory prognosis in GC patients. SLC6A14 depletion held the EMT back to confer reduced GC metastasis for which amino acid starvation was triggered, followed by the mitigated activation of the PI3K/AKT/mTORC1 pathway. Taken together, SLC6A14 might be a druggable target in the treatment of metastatic GC.

## Figures and Tables

**Table 1:** Correlation between SLC6A14 expression and the clinicopathological features of gastric cancers.

Clinicopathological features	*N*	SLC6A14 expression				*P* value
		Low	High	PR (%)		
		***+***	***++***	***+++***		
*Age*						0.30
<50years	53	18	15	20	66.8	
≥50 years	50	21	10	19	59.3	
*Gender*						0.71
Male	48	19	14	15	60.1	
Female	55	20	15	20	63.2	
*Distant metastasis*						0.009
M0	52	36	7	9	30.2	
M1	51	3	16	32	94.0	
*Depth of invasion*						0.023
Tis-1	23	20	1	2	13.09	
T2	25	9	10	6	62.0	
T3	29	5	19	5	82.6	
T4	26	5	10	11	80.0	
*Venous invasion*						0.033
—	51	24	13	14	50.1	
+	52	15	29	8	71.4	
*Lymph node metastasis*						0.035
N0	24	18	3	3	25.2	
N1	26	12	11	3	53.4	
N2	28	4	18	6	85.5	
N3	25	5	12	8	86.0	
*Lymphatic invasion*						0.021
—	48	25	16	7	41.5	
+	55	14	24	17	74.5	
*Tumor differentiation*						0.75
Well or moderate	50	20	18	12	60.0	
Poor	53	19	18	16	64.0	
*AJCC staging*						0.65
Stage I	25	18	4	3	28.0	
Stage II	26	12	10	4	53.8	
Stage III	24	6	12	6	75.0	
Stage IV	28	3	10	15	89.3	

PR (%): positive rate; *χ*^2^ test was executed to assess the association between SLC6A14 expression and the pathological features of GC patients. ^∗^*P* < 0.05,  ^∗∗^*P* < 0.01, and^#^*P* > 0.05.

## Data Availability

The data used to support the findings of this study are available.

## Supplementary Materials

Supplementary Materials Supplemental Materials and Methods. High-Content Screening. Supplementary Fig. S1: upregulated mRNAs (^∗^*P* < 0.05, FC ≥ 3.0) in MKN28-M (A) and SGC7901-M (B), as compared with MKN-28-NM and SGC7901-NM cells, respectively. These transcripts were sequenced from high to low by a multiple of fold change. Supplementary Fig. S2: the details of the DEGs that enriched in the PI3K signaling pathway in MKN28-M cells with SLC6A14 knockdown were shown.
