# Supplementary material for: SLC6A14 Depletion Contributes to Amino Acid Starvation to Suppress EMT-Induced Metastasis in Gastric Cancer by Perturbing the PI3K/AKT/mTORC1 Pathway
Source: Biomed Res Int. 2022 Jul 12;2022:7850658. doi: 10.1155/2022/7850658 (PMC9296317; doi:10.1155/2022/7850658)
Supplement: Supplementary Materials — Supplemental Materials and Methods. High-Content Screening. Supplementary Fig. S1: upregulated mRNAs (∗P < 0.05, FC ≥ 3.0) in MKN28-M (A) and SGC7901-M (B), as compared with MKN-28-NM and SGC7901-NM cells, respectively. These transcripts were sequenced from high to low by a multiple of fold change. Supplementary Fig. S2: the details of the DEGs that enriched in the PI3K signaling pathway in MKN28-M cells with SLC6A14 knockdown were shown. [file 7850658.f1.zip › Supplementary Fig. S1B.pdf]

Gene Set Name	Gene Symbol	Description	Fold Change
GO_SMALL_MOLECULE_METABOLIC_PROCESS	**SLC6A14**	solute carrier family 6 (amino acid transporter), member 14	8.1399564
GO_ORGANIC_ACID_METABOLIC_PROCESS	**SLC6A14**	solute carrier family 6 (amino acid transporter), member 14	8.1399564
GO_NEGATIVE_REGULATION_OF_PROTEIN_METABOLIC_PROCESS	**CST1**	cystatin SN	6.4383819
GO_NEGATIVE_REGULATION_OF_MOLECULAR_FUNCTION	**CST1**	cystatin SN	6.4383819
GO_NEGATIVE_REGULATION_OF_CATALYTIC_ACTIVITY	**CST1**	cystatin SN	6.4383819
GO_NEGATIVE_REGULATION_OF_PROTEIN_METABOLIC_PROCESS	**SPINK4**	serine peptidase inhibitor, Kazal type 4	6.0240295
GO_NEGATIVE_REGULATION_OF_MOLECULAR_FUNCTION	**SPINK4**	serine peptidase inhibitor, Kazal type 4	6.0240295
GO_NEGATIVE_REGULATION_OF_CATALYTIC_ACTIVITY	**SPINK4**	serine peptidase inhibitor, Kazal type 4	6.0240295
GO_NEGATIVE_REGULATION_OF_RESPONSE_TO_STIMULUS	**RGS2**	Regulator of G-protein signaling 2	5.9979263
GO_NEGATIVE_REGULATION_OF_CELL_COMMUNICATION	**RGS2**	Regulator of G-protein signaling 2	5.9979263
GO_INTRACELLULAR_SIGNAL_TRANSDUCTION	**RGS2**	Regulator of G-protein signaling 2	5.9979263
GO_REGULATION_OF_MULTICELLULAR_ORGANISMAL_DEVELOPMENT	**RGS2**	Regulator of G-protein signaling 2	5.9979263
GO_REGULATION_OF_CELL_PROLIFERATION	**RGS2**	Regulator of G-protein signaling 2	5.9979263
GO_IMMUNE_SYSTEM_PROCESS	**RGS2**	Regulator of G-protein signaling 2	5.9979263
GO_POSITIVE_REGULATION_OF_RESPONSE_TO_STIMULUS	**RGS2**	Regulator of G-protein signaling 2	5.9979263
GO_RESPONSE_TO_OXYGEN_CONTAINING_COMPOUND	**RGS2**	Regulator of G-protein signaling 2	5.9979263
GO_CELL_CYCLE	**RGS2**	Regulator of G-protein signaling 2	5.9979263
GO_POSITIVE_REGULATION_OF_GENE_EXPRESSION	**PGM2L1**	phosphoglucomutase 2-like 1	5.7820756
GO_NEGATIVE_REGULATION_OF_RESPONSE_TO_STIMULUS	**PGM2L1**	phosphoglucomutase 2-like 1	5.7820756
GO_NEGATIVE_REGULATION_OF_CELL_COMMUNICATION	**PGM2L1**	phosphoglucomutase 2-like 1	5.7820756
GO_NEGATIVE_REGULATION_OF_PROTEIN_METABOLIC_PROCESS	**PGM2L1**	phosphoglucomutase 2-like 1	5.7820756
GO_INTRACELLULAR_SIGNAL_TRANSDUCTION	**GATA6**	GATA binding protein 6	5.5820756
GO_REGULATION_OF_MULTICELLULAR_ORGANISMAL_DEVELOPMENT	**GATA6**	GATA binding protein 6	5.5820756
GO_TISSUE_DEVELOPMENT	**GATA6**	GATA binding protein 6	5.5820756
GO_NEGATIVE_REGULATION_OF_GENE_EXPRESSION	**GATA6**	GATA binding protein 6	5.5820756
GO_POSITIVE_REGULATION_OF_BIOSYNTHETIC_PROCESS	**SAMD13**	sterile alpha motif domain containing 13	5.3520756
GO_REGULATION_OF_PROTEIN_MODIFICATION_PROCESS	**SAMD13**	sterile alpha motif domain containing 13	5.3520756
GO_REGULATION_OF_CELL_PROLIFERATION	**SAMD13**	sterile alpha motif domain	5.3520756
GO_REGULATION_OF_RESPONSE_TO_STRESS	**SAMD13**	sterile alpha motif domain	5.3520756
GO_NEGATIVE_REGULATION_OF_NITROGEN_COMPOUND_METABOLIC_PROCESS	**SAMD13**	sterile alpha motif domain containing 13	5.3520756
GO_INTRACELLULAR_SIGNAL_TRANSDUCTION	**ARL2**	ADP-ribosylation factor-like 2	5.2148959
GO_NEGATIVE_REGULATION_OF_MOLECULAR_FUNCTION	**ARL2**	ADP-ribosylation factor-like 2	5.2148959
GO_NEGATIVE_REGULATION_OF_CATALYTIC_ACTIVITY	**ARL2**	ADP-ribosylation factor-like 2	5.2148959
GO_PROTEIN_COMPLEX_SUBUNIT_ORGANIZATION	**ARL2**	ADP-ribosylation factor-like 2	5.2148959
GO_MACROMOLECULAR_COMPLEX_ASSEMBLY	**ARL2**	ADP-ribosylation factor-like 2	5.2148959
GO_CELL_CYCLE	**ARL2**	ADP-ribosylation factor-like 2	5.2148959
GO_REGULATION_OF_PROTEIN_MODIFICATION_PROCESS	**ACY3**	aminoacylase 3	5.1180169
GO_IMMUNE_SYSTEM_PROCESS	**ACY3**	aminoacylase 3	5.1180169
GO_POSITIVE_REGULATION_OF_RESPONSE_TO_STIMULUS	**ACY3**	aminoacylase 3	5.1180169
GO_INTRACELLULAR_SIGNAL_TRANSDUCTION	**ADAMTSL**	ADAMTS like 4	4.9406543
GO_REGULATION_OF_CELL_PROLIFERATION	**ADAMTSL**	ADAMTS like 4	4.9406543
GO_REGULATION_OF_RESPONSE_TO_STRESS	**ADAMTSL**	ADAMTS like 4	4.9406543
GO_IMMUNE_SYSTEM_PROCESS	**ADAMTSL**	ADAMTS like 4	4.9406543

Supplementary Figure 1B
