# Supplementary material for: SLC6A14 Depletion Contributes to Amino Acid Starvation to Suppress EMT-Induced Metastasis in Gastric Cancer by Perturbing the PI3K/AKT/mTORC1 Pathway
Source: Biomed Res Int. 2022 Jul 12;2022:7850658. doi: 10.1155/2022/7850658 (PMC9296317; doi:10.1155/2022/7850658)
Supplement: Supplementary Materials — Supplemental Materials and Methods. High-Content Screening. Supplementary Fig. S1: upregulated mRNAs (∗P < 0.05, FC ≥ 3.0) in MKN28-M (A) and SGC7901-M (B), as compared with MKN-28-NM and SGC7901-NM cells, respectively. These transcripts were sequenced from high to low by a multiple of fold change. Supplementary Fig. S2: the details of the DEGs that enriched in the PI3K signaling pathway in MKN28-M cells with SLC6A14 knockdown were shown. [file 7850658.f1.zip › Supplementary Fig. S2.pdf]

Gene Set Name	Gene Symbol	Description	Fold Change
KEGG_PI3K_SIGNALING_PATHWAY	NFKBIB	nuclear factor of kappa light polypeptide gene enhancer in B-cells inhibitor, beta	2.97617108
KEGG_PI3K_SIGNALING_PATHWAY	PRAS40	proline-rich Akt substrate of 40 kDa	2.90332322
KEGG_PI3K_SIGNALING_PATHWAY	GSK3B	glycogen synthase kinase 3 beta	2.03904033
KEGG_PI3K_SIGNALING_PATHWAY	GSK3A	glycogen synthase kinase 3 alpha	1.89943985
KEGG_PI3K_SIGNALING_PATHWAY	GSKIP	GSK3B interacting protein	1.87483493
KEGG_PI3K_SIGNALING_PATHWAY	PIK3CA	phosphatidylinositol-4,5-bisphosphate 3-kinase, catalytic subunit alpha	-1.32232232
KEGG_PI3K_SIGNALING_PATHWAY	PIK3R3	phosphoinositide-3-kinase, regulatory subunit 3 (gamma)	-1.3232325
KEGG_PI3K_SIGNALING_PATHWAY	PIK3R4	phosphoinositide-3-kinase, regulatory subunit 4	-1.54592652
KEGG_PI3K_SIGNALING_PATHWAY	PIK3C2A	phosphatidylinositol-4-phosphate 3-kinase, catalytic subunit type 2 alpha	-1.67602594
KEGG_PI3K_SIGNALING_PATHWAY	NKRF	NFKB repressing factor	-1.74063167
KEGG_PI3K_SIGNALING_PATHWAY	PIK3C2G	phosphatidylinositol-4-phosphate 3-kinase, catalytic subunit type 2 gamma	-1.78323233
KEGG_PI3K_SIGNALING_PATHWAY	PIK3IP1	phosphoinositide-3-kinase interacting protein 1	-1.83232323
KEGG_PI3K_SIGNALING_PATHWAY	NFKB1	nuclear factor of kappa light polypeptide gene enhancer in B-cells 1	-1.87382393
KEGG_PI3K_SIGNALING_PATHWAY	PIK3CG	phosphatidylinositol-4,5-bisphosphate 3-kinase, catalytic subunit gamma	-1.87832992
KEGG_PI3K_SIGNALING_PATHWAY	PIK3CB	phosphatidylinositol-4,5-bisphosphate 3-kinase, catalytic subunit beta	-1.8790302
KEGG_PI3K_SIGNALING_PATHWAY	PIK3R5	phosphoinositide-3-kinase, regulatory subunit 5	-1.98329389
KEGG_PI3K_SIGNALING_PATHWAY	NFKBIA	nuclear factor of kappa light polypeptide gene enhancer in B-cells inhibitor, alpha	-1.98392832
KEGG_PI3K_SIGNALING_PATHWAY	PIK3CD	phosphatidylinositol-4,5-bisphosphate 3-kinase, catalytic subunit delta	-2.01253849
KEGG_PI3K_SIGNALING_PATHWAY	MTOR	Mechanistic target of rapamycin (serine/threonine kinase)	-2.23229899
KEGG_PI3K_SIGNALING_PATHWAY	PIK3C2B	phosphatidylinositol-4-phosphate 3-kinase, catalytic subunit type 2 beta	-2.32323544
KEGG_PI3K_SIGNALING_PATHWAY	AKT3	v-akt murine thymoma viral oncogene homolog 3	-2.32389933
KEGG_PI3K_SIGNALING_PATHWAY	NFKB2	nuclear factor of kappa light polypeptide gene enhancer in B-cells 2 (p49/p100)	-2.32899849
KEGG_PI3K_SIGNALING_PATHWAY	4EBP1	Eukaryotic translation initiation factor 4E binding protein 1	-2.73323232
KEGG_PI3K_SIGNALING_PATHWAY	MLST8	MTOR associated protein, LST8 homolog	-2.78392939
KEGG_PI3K_SIGNALING_PATHWAY	p70S6K	Ribosomal protein S6 kinase,S6K1	-2.93829393
KEGG_PI3K_SIGNALING_PATHWAY	Raptor	regulatory associated protein of mTOR	-2.98399232
KEGG_PI3K_SIGNALING_PATHWAY	AKT2	v-akt murine thymoma viral oncogene homolog2	-3.23232326
KEGG_PI3K_SIGNALING_PATHWAY	DEPDC6	DEP domain-containing protein 6	-3.43434255
KEGG_PI3K_SIGNALING_PATHWAY	AKT1	v-akt murine thymoma viral oncogene homolog 1	-3.43434343
KEGG_PI3K_SIGNALING_PATHWAY	EIF-2α	Eukaryotic translation initiation factor 2 alpha	-3.78793239
KEGG_PI3K_SIGNALING_PATHWAY	PIK3R1	phosphoinositide-3-Kinase Regulatory Subunit 1	-4.32323232

Supplementary Figure 2
