# Supplementary material for: SLC6A14 Depletion Contributes to Amino Acid Starvation to Suppress EMT-Induced Metastasis in Gastric Cancer by Perturbing the PI3K/AKT/mTORC1 Pathway
Source: Biomed Res Int. 2022 Jul 12;2022:7850658. doi: 10.1155/2022/7850658 (PMC9296317; doi:10.1155/2022/7850658)
Supplement: Supplementary Materials — Supplemental Materials and Methods. High-Content Screening. Supplementary Fig. S1: upregulated mRNAs (∗P < 0.05, FC ≥ 3.0) in MKN28-M (A) and SGC7901-M (B), as compared with MKN-28-NM and SGC7901-NM cells, respectively. These transcripts were sequenced from high to low by a multiple of fold change. Supplementary Fig. S2: the details of the DEGs that enriched in the PI3K signaling pathway in MKN28-M cells with SLC6A14 knockdown were shown. [file 7850658.f1.zip › Supplementary Materials and Methods.docx]

**Supplemental Materials and methods**

***High-Content Screening***

*High-Content Screening* were conducted according to the methods demonstrated in the

***Materials and methods.*** The 2-day cell migration rate was analyzed using the following formula:


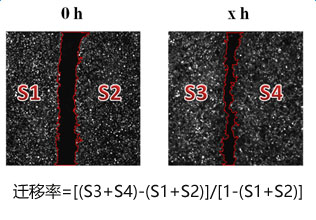


Migration rate= [(S3+S4)-(S1+S2)]/(1-(S1+S2)] .S1, S2,S3 and S4 represented the cell area(%).
